# Twelve-Hour Ultrarush Immunotherapy in a Patient With Mastocytosis and Hymenoptera Sting Anaphylaxis

**DOI:** 10.1097/WOX.0b013e31819b0413

**Published:** 2009-03-15

**Authors:** Doris Jäger, Jürgen Barth

**Affiliations:** 1 Practice of Internal and Pulmonary Diseases and Medical Clinic Berufsgenossenschaftliche Kliniken Bergmannstrost Halle/Saale, Germany

## To the Editor

Mastocytosis (MC) denotes a heterogeneous group of clinical disorders caused by abnormal growth and accumulation of mast cells (incidence in the general population presumably 7 new cases per 1,000,000 persons each year). It ranges from primarily cutaneous manifestations of urticaria pigmentosa to systemic mastocytosis (SM), in which virtually every tissue can be involved, to rare malignant forms such as mast cell leukemia [1-3]. A well-established serum marker of SM is tryptase (α- and β-tryptase forms). The current assays are thought to measure pro-tryptase, resulting from continuous release from mast cells in various organs (reference range, < 1-11.4 ng/mL in our system) [1-3]. In SM, tryptase levels normally exceed 20 ng/mL. But as tryptase can also be elevated because of other diseases (eg, myelodysplastic syndrome), it is only a minor criterion for SM [1,3,4].

Patients with SM are overrepresented in the group of persons with anaphylaxis to Hymenoptera venom (prevalence between 1% and 2.6%) [1,2,5]. As type 1 sensitization to venoms detected by skin test or serology is reported to be comparatively low in some studies,[1,4-8] additional immunoglobulin E (IgE)-independent pathways are thought to be important: direct action of histamine-liberating or complement-activating substances present in venom such as mast cell-degranulating peptide, mellitin, or phospholipases [6]. In contrast to this, Ruëff et al [2] could demonstrate comparable specific venom sensitization in patients after insect anaphylaxis with and without MC.

Generally, MC patients are endangered by life-threatening reactions after insect stings secondary to increased mediator release [1,2,5-7]. Therefore, specific venom immunotherapy (VIT), the only available causal treatment of insect venom hypersensitization so far, is urgently recommended. On the other hand, these patients tend to express more serious side effects,[1,2,5,7-9] in some cases, VIT even had to be stopped prematurely [1,7]. In addition to this, Dubois [1] and the group of Ruëff et al [2] found an increased rate of sting reactions during or after VIT in MC patients, even with fatal outcomes [9,10]. These data suggest that the efficacy of VIT may be reduced in patients with mastocytosis, particularly in vespid-allergic patients [1,2,4]. As ultrarush or rapid VIT (RVIT) has been proven reliable and efficacious with a low incidence of systemic reactions,[11,12] we chose this protocol for an MC patient with wasp venom anaphylaxis.

The 41-year-old female patient had histologically confirmed cutaneous mast cell disease with typical urticaria pigmentosa. Tryptase level was slightly elevated to 16.3 ng/mL(average, about 5 ng/mL in controls), so that indolent SM was also discussed, but a bone marrow biopsy was not feasible for confirmation.

In September 2003, she experienced an anaphylactic reaction grade III to IV, including local and remote skin reactions according to Mueller HL,[13] lightheadedness, hypotension, and dyspnea after a wasp sting. Although being informed about the potentially higher risk, she explicitly selected RVIT in January 2004 to reduce hospitalization time.

Four months later, she was checked for allergy status. Total serum IgE was less than 10 IU/mL. The patient had no history of atopy, and prick tests with perennial and seasonal aeroallergens were negative. Bee venom remained negative in prick test until 300 μg/mL, whereas vespula venom was positive here at 0.1 μg/mL.

Intracutaneous tests were performed with serial 10-fold dilutions of vespula venom. The end point concentration still resulting in a positive reaction here was 0.00001 μg/mL. The initial wasp-specific IgE was 2 kU/L (class 2) (CAP Test, Fa. Phadia, Sweden), and specific IgG resulted in 11.5 mg/L (= intermediately elevated).

The VIT followed a modified ultrarush protocol [14] (Table 1), with a subcutaneous injection of 0.00001 μg wasp venom (Venomil wasp, Fa. Bencard, Munich, Germany) and increasing doses 10-fold every 30 minutes until 10 μg, followed by 20, 30, 50, and 100 μg in 30-minute intervals. The maximum single dose of 100 μg was reached after 12 hours with only local side effects.

**Table 1:** Rapid VIT Treatment Protocol for Updosing of Wasp Venom in Our Patient With MC and Clinical Data

Time, h	Delivered Dose of Wasp Venom, μg	Adverse Reactions	Blood Pressure, mm Hg; Heart Rate, min
0	0.00001	None	128/80; 109
0.5	0.0001	None	138/79; 108
1	0.001	None	131/90; 107
1.5	0.01	None	120/88; 101
2	0.1	Mild erythema, 1 cm	129/79; 114
2.5	1	Wheal and flare, 3 cm	116/86; 116
3	10	Mild erythema, 1 cm	137/87; 123
4	20	None	106/67; 106
5	30	None	114/72; 109
6	50	None	112/62; 103
12	100	Erythema, 2 cm	115/81; 99

The patient was pretreated with intravenous dimetindene 6 mg just before VIT. On day 2, she was discharged. Booster injections of 100 μg were given at 7, 14, and 21 days, and then every 4 weeks on an outpatient basis.

**Figure 1:**
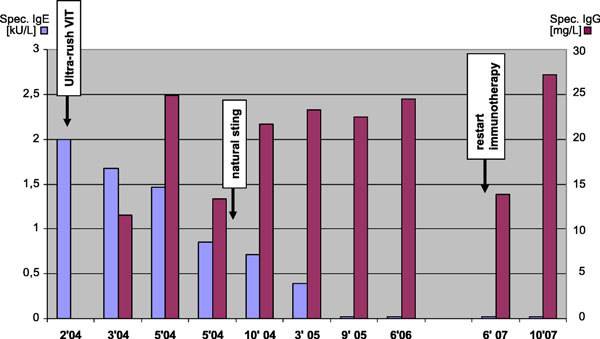
**Titers of specific wasp venom IgE and IgG antibodies during the course of therapy**. The VIT was interrupted for 1 year because of pregnancy, and then restarted, without systemic side effects.

In Figure 1, both the increase of wasp-specific IgG and the continuous decrease of specific IgE from class 2 to below detection limit after 18 months of therapy are presented. In August 2004, the patient has tolerated a natural wasp sting with only local symptoms.

No severe side effects were seen over a treatment period of more than 2 years until VIT was discontinued in July 2006 because of pregnancy on the advice of the gynecologist. The patient now continues to do well after VIT has been restarted with a short modified rush of wasp venom from 0.01 to 100 μg for 4 hours. Interestingly, specific IgE had still been very low after 1 year of cessation of treatment, but specific IgG had dropped to almost the initial level. It increased again to 27.2 mg/L after 4 months of treatment (Figure 1).

As classic IgE-mediated allergic reactions are under-represented in patients with MC combined with insect hyperreactivity,[1,4-7] it was speculated that binding of total and specific IgE to abundant tissue mast cells could be responsible for low serum levels [7,15]. Many authors decided to perform immunotherapy anyhow, independent of allergy test results, because even if serology is negative, significant levels of venom specific IgE may still be present at the mast cell and tissue level [6-8]. In some patients, MC is first diagnosed through a life-threatening insect sting anaphylaxis,[5,7] and other therapeutical options are not at hand.

Conventional VIT normally reaches the maintenance dose of about 100 μg or cumulative doses between 100 and 500 μg venom protein after several weeks to months, whereas rush-regimen use schedules between 5 and 10 days [8,11,12]. The first ultrarush protocol dates back to 1983, where Van der Zwan et al [14] performed a hyposensitization to wasp venom within 6 hours in 11 patients without systemic reaction. Ultrarush or rapid VIT use induction schemes between 90 minutes and 2 days [11,12]. So far, no standard rapid or ultrarush VIT protocol has been widely adopted. Patients with a positive sting challenge after VIT may be protected by increased maintenance dose [2]. Some authors propose lifelong VIT in MC because of the higher risk of severe relapses [2,4].

Rapid VIT was well tolerated in our case and provided protection against a field sting. We documented a decrease of specific IgE and increase in specific IgG, which has a proposed blocking antibody mechanism [14].

The rationale for us to choose RVIT was the results of McHugh et al,[16] who demonstrated a rapid decrease of interleukin 4 during the first treatment hours and a shift from Th2 to a Th1 pattern, which was interpreted as an early mechanism of immune modulation.

In summary, with this case, we present for the first time an ultrarush specific immunotherapy (= rapid VIT) within 12 hours in a patient with histologically confirmed MC after anaphylactic reaction to a vespid sting. We concluded that even in these high-risk patients, RVIT could serve as a safe and effective treatment, reducing the cost and time commitment, promising a favorable patient compliance. Although it must be pointed out that the tolerance of ultrarush VIT in a larger series of MC patients, especially with proven systemic form or with more severe sting anaphylaxis, is still unknown, and therefore, caution is indispensable. The optimal duration and maintenance dosage of RVIT for MC patients need further evaluation as well.

Doris Jäger, MD

Jürgen Barth, MD, PhD

Practice of Internal and Pulmonary Diseases and Medical Clinic Berufsgenossenschaftliche Kliniken Bergmannstrost Halle/Saale, Germany
